# A simplified LC–MS-based method for sensitive analysis of DNA adducts utilizing accessible in vitro metabolism models

**DOI:** 10.1007/s00204-025-04125-w

**Published:** 2025-07-08

**Authors:** Andrea Gerdemann, Matthias Behrens, Georgia Günther, Ahmed Ghallab, Jan G. Hengstler, Hans-Ulrich Humpf, Melanie Esselen

**Affiliations:** 1 Institute of Food Chemistry, University of Münster, Corrensstraße 45, 48149 Münster, Germany; 2 Leibniz Research Centre for Working Environment and Human Factors (IfADo), Ardeystraße 67, 44139 Dortmund, Germany; 3 Department of Forensic Medicine and Toxicology, Faculty of Veterinary Medicine, South Valley University, Qena, 83523 Egypt

**Keywords:** Alpha-asarone and beta-asarone, Methyleugenol, 3-Monochloropropanediol, Benzo[a]pyrene, Aflatoxin B_1_, Liver S9 fraction, HepG2 cells, Primary hepatocytes

## Abstract

Genetic information of living cells is encoded by the specific arrangement of nucleobases in deoxyribonucleic acid (DNA). Even minor changes or modifications of these nucleobases, favored by many reactive sites, can significantly affect correct replication and DNA integrity leading to severe toxic effects. Analyzing these DNA modifications is highly complex and often requires non-specific assays for DNA damage or ^32^*P*-postlabelling. A major limitation of these methods is their inability to provide structural information, a gap that can be addressed by instrumental analytical techniques. An additional major challenge is the selection of an appropriate biological system capable of reliable DNA adduct formation in high yields, since most compounds require metabolic activation prior to reacting with DNA. Therefore, the aim of this study was to develop a fast and simple workflow for sensitive DNA adduct analysis. In addition to highlighting the main pitfalls in sample preparation, this publication focuses on the comparison of biological systems in terms of metabolic activity, using six well-known carcinogens from different chemical classes. The combination of HepG2 cells and liver S9 fractions demonstrated comparable or even superior capabilities to primary hepatocytes and enabled the detection of DNA adducts from aflatoxin B_1_, benzo[a]pyrene, methyleugenol as well as α-asarone and β-asarone, particularly after metabolic activation by the aryl hydrocarbon receptor agonist β-naphthoflavone. Notably, the adduct formation of phenylpropanoids was documented for the first time in a non-transfected cancer cell line using high-performance liquid chromatography coupled to mass spectrometry. Therefore, the method can be used to detect previously unknown DNA adducts from diverse chemical classes and provides structural insights into the formed DNA adducts.

**Supplementary Information:**

The online version contains supplementary material available at 10.1007/s00204-025-04125-w.

## Introduction

The genetic information of living organisms is stored as deoxyribonucleic acid (DNA) (Avery et al. 1944). DNA is made up of four nucleosides, each consisting of a purine or pyrimidine base linked to a deoxyribose. These monomers are connected by phosphodiester bonds to form a macromolecular helix (Watson and Crick 1953). Purine and pyrimidine bases contain different nucleophilic sites, which can bind electrophilic xenobiotics leading to modified genetic information. Chemical modification depends on nucleophilicity as well as the steric factors of DNA components. Therefore, the endocyclic *N*−3 and *N*−7 positions of guanine (Gua) and adenine (Ade) are preferentially bound by xenobiotics. The *N*−7 positions are more accessible for bulky xenobiotics as they are localized in the major groove of the DNA double helix. Because of the formal positive charge of *N*−7 Gua adducts, those are chemically unstable leading to base-catalyzed rearrangement to formamidopyrimidine adducts (FAPY) or depurination. Highly electrophilic substances can also bind to the less nucleophilic *N*−2 positions of Gua. Additionally, *O*−6 position of guanine or *O*−4 and *O*−2 position of thymidine as well as *O*−2 position of cytosine are often alkylated (La and Swenberg 1996; Bedard and Massey 2006; Boysen et al. 2009).

As the formation of DNA adducts is involved in mutagenicity and the initiation of carcinogenesis, the analysis of DNA adduct formation is especially important for newly discovered xenobiotics. Formation of DNA adducts is traditionally determined via indirect methods providing information about DNA damage, mutagenicity or genotoxicity, but not directly on DNA adduct formation and structure. In contrast, mass spectrometric methods enable sensitive determination of DNA adducts while simultaneously providing structural information on the formed adducts. Because of the repetitive structural components, unknown DNA adducts can be identified easily based on their typical fragmentation pattern cleaving the carbohydrate moiety or the DNA base. Therefore, the occurrence of the protonated purine or pyrimidine fragment ions or the neutral loss of 116 (deoxyribose) in the mass spectra enable unequivocal identification of DNA adducts without reference compounds (Balbo et al. 2014).

To study the DNA binding properties of chemicals, usually metabolizing systems are required, because only relatively few compounds bind to the DNA directly. Most xenobiotics are metabolized by cytochrome P450 monooxygenases (CYP) introducing or unmasking functional groups to increase their aqueous solubility. Also phase II conjugation, such as sulfation or glucuronidation, is involved in xenobiotic metabolism enabling easier excretion. Instead of leading to detoxification and excretion, these reactions can also induce their bioactivation and subsequent DNA binding (Guengerich 2008; Jancova et al. 2010). To simulate these metabolic reactions, liver S9 fractions, primary hepatocytes or cancer cell lines are used in research.

Each of these models has distinct advantages and limitations regarding their use in DNA adductomics, which were compared and evaluated in this study. Many of the current approaches using high-performance liquid chromatography with mass spectrometric detection (HPLC–MS) focus on a small number of specific DNA adducts, whereas untargeted methods capable of detecting unknown adducts are still scarce (Tang and Zhang 2020). Therefore, the development of a sensitive and comprehensive HPLC–MS method and the use of a readily available metabolic activation method were major objectives of this study. For this reason, the metabolic competences of three individual cancer cell lines were compared and optimized using aflatoxin B_1_ (AFB_1_) as a model compound. Subsequently, a comprehensive comparison of HepG2 cells with murine primary hepatocytes and mammalian liver S9 fractions was conducted using structurally diverse compounds that exhibit well-known DNA-binding properties.

## Materials and methods

### Chemicals and reagents

All chemicals were purchased from Carl Roth (Karlsruhe, Germany), Sigma-Aldrich (Steinheim, Germany) or VWR (Darmstadt, Germany) if not further specified. Solvents were used in LC–MS grade. All chemicals and consumables used for DNA isolation were sterilized prior to their use to inactivate DNases and reduce DNA degradation. Aflatoxin B_1_-*N-*7-guanine (AFB_1_-Gua) was synthesized as reported previously (Gerdemann et al. 2023).

### Cell culture

HepG2 cells (ATCC, Manassas, USA), HT-29 cells (ATCC, Manassas, USA) and A549 cells (DSMZ, Braunschweig, Germany) were cultured in Dulbecco’s Modified Eagle Medium (DMEM, high glucose, Gibco, Prat de Lloregat, Barcelona, Spain) supplemented with 10% fetal calf serum, 10 mM *N*−2-hydroxyethylpiperazine-*N*′−2-ethanesulfonic acid (HEPES, Carl Roth, Karlsruhe, Germany) and two antibiotics penicillin (100 U/mL) and streptomycin (100 µg/mL) obtained from PAN Biotech (Aidenbach, Germany).

Primary rat and mouse hepatocytes (prH and pmH) from male Wistar rats and male C57BL/6N mice were cultured in collagen sandwiches with supplemented Williams E medium as described previously (Gerdemann et al. 2023).

### Metabolic activation via β-NF preincubation

HepG2 cells were seeded in cell culture dishes with a 10 cm diameter at a density of 3 × 10^6^ cells/dish. After 24 h, the medium was replaced by serum-free medium containing 1 µM β-naphthoflavone (β-NF), 10 µM β-NF, 100 µM β-NF to induce CYP expression or 1% dimethyl sulfoxide as solvent control, respectively. After 24 h the medium was replaced by Hank’s balanced salt solution (HBSS, PAN Biotech, Aidenbach, Germany) containing 10 µM AFB_1_ for an additional 24 h. HBSS was used instead of DMEM to reduce matrix effects in medium analysis. Afterwards, the medium was removed and concentrated under vacuum by a factor of 10. Furthermore, DNA was isolated from the cells, digested and analyzed via high-performance liquid chromatography coupled to tandem-mass spectrometry (HPLC–MS/MS). The experiment was performed in three independent cell passages with two seeding replicates each.

### Comparison of cell lines

Cancer cell lines were seeded in cell culture dishes with a 10 cm diameter at a density of 3 × 10^6^ cells/dish. The medium was replaced by serum-free medium supplemented with 10 µM β-NF after 24 h. Another 24 h later the medium was replaced by HBSS containing 10 µM AFB_1_. In addition to AFB_1_, glycidamide (GA) was tested as described in Online Resource 1. After 24 h of treatment, cells were lysed, DNA was extracted, digested and all samples were analyzed via HPLC–MS/MS. The experiment was performed in three independent cell passages with two seeding replicates each.

### DNA isolation and digestion procedure in cell culture experiments

The cells were washed twice with phosphate-buffered saline (PBS, PAN Biotech, Aidenbach, Germany) and scraped with 500 µL lysis buffer (200 mM tris(hydroxymethyl)aminomethane (TRIS), pH 8.5, 250 mM sodium chloride, 25 mM ethylenediaminetetraacetic acid, 0.5% sodium dodecyl sulfate). The solution was transferred into a DNase-free reaction tube and resuspended rigorously. After lysing the cells, 5 µL ribonuclease (10 mg/mL) was added and samples were shaken over 5 min followed by the addition of 20 µL proteinase K (2 mg/mL) and 5 min gentle shaking. The digestion times must be strictly adhered to reduce analyte degradation. Afterwards, 300 µL potassium acetate (5 M, pH 7) was added to precipitate remaining proteins and neutralize phosphate backbone to enable DNA precipitation (Green and Sambrook 2016). After centrifugation (14,840 × *g*, 30 min, 4 °C), the supernatant was mixed with 1 mL pre-cooled isopropanol. Samples were inverted gently until the DNA precipitated clearly. After centrifugation (14,840 × *g*, 30 min, 4 °C), the supernatant was removed and the DNA residue was washed with 500 µL 70% ethanol. The samples were centrifuged (14,840 × *g*, 30 min, 4 °C), the precipitate dried under vacuum and dissolved in 100 µL digestion buffer containing 2 U DNase, 100 mU phosphodiesterase and 500 mU alkaline phosphatase, dissolved in a buffer of 10 mM TRIS and 5 mM MgCl_2_ (pH 7). The digestion was carried out overnight at 37 °C under gentle shaking and was stopped by adding 300 µL acetonitrile. After centrifugation (14,840 × *g*, 15 min, 4 °C), the supernatant was dried under vacuum and re-dissolved in 50 µL water followed by HPLC–MS analysis. An aliquot of the solution was diluted 100 times in water for data normalization to the deoxyguanosine (dG) content.

### Comparison of HepG2 cells, primary hepatocytes and liver S9 fractions

HepG2 cells were seeded at a density of 9 × 10^6^ cells/dish in cell culture dishes with 10 cm diameter. Cells were cultured for seven days in serum-containing medium and afterwards preincubated with 10 µM β-NF in serum-containing medium over 24 h followed by compound treatment (1 mM 3-monochloropropanediol (3-MCPD), 10 µM benzo[a]pyrene (B[a]P), 500 µM methyleugenol (ME), 100 µM α-asarone (α-AS), 100 µM β-asarone (β-AS) or 10 µM AFB_1_) in HBSS. Their chemical structures are shown in Online Resource 1 (Fig. S1). In this experiment, higher cell counts were used to increase the conversion rate of the compounds as well as the amount of DNA for higher sensitivity. After 24 h, DNA was isolated, digested and analyzed via high-performance liquid chromatography coupled to high-resolution mass spectrometry (HPLC-HRMS) and HPLC–MS/MS. The experiment was performed in three seeding replicates for each compound.

Primary hepatocytes were seeded in 6-well plates at a density of 0.85 × 10^6^ cells/well (mouse) and 1 × 10^6^ cells/well (rat) and treated with the respective compounds (except 3-MCPD) at the same concentrations as the HepG2 cells. Due to fast DNA repair, the incubation time was reduced to 4 h. In the experiment with AFB_1_ the incubation period was 2 h. After incubation, cells were washed with PBS and scraped with 200 µL of lysis buffer. DNA was extracted and digested as described previously (Gerdemann et al. 2023). For reasons of availability, the experiment was performed with two seeding replicates.

Porcine and equine liver S9 fractions were prepared with modifications according to Hoensch et al. (1985). Compounds were added at a concentration of 100 µM to a suspension of 100 mM phosphate buffer (pH 7.5), 0.5 mg/mL calf thymus DNA, 10 mM glucose 6-phosphate, 664 µM nicotinamide adenine dinucleotide phosphate, 2 U/mL glucose 6-phosphate dehydrogenase and 3 mg/mL liver S9 fraction. After 24 h, the reaction was stopped by adding 200 µL lysis buffer to the 100 µL reaction suspension. Subsequently, 150 µL potassium acetate (5 M, pH 7) was added and the samples were centrifuged (14,840 × *g*, 30 min, 4 °C). The supernatant was mixed with 500 µL of pre-cooled isopropanol to precipitate the DNA and centrifuged again (14,840 × *g*, 30 min, 4 °C). From this point, samples were treated identical to cell culture samples.

### HPLC-HRMS and HPLC–MS/MS analysis

DNA adducts were separated on a Nucleodur C18 Pyramid column (Macherey-Nagel, Düren, Germany, 150 mm × 2 mm, 3 µm) with a gradient of acetonitrile and water, supplemented with 0.1% formic acid each, and a flow rate of 0.5 mL/min. The gradient started with 2 min of 100% aqueous solvent to separate even very polar compounds. The aqueous solvent was reduced to 5% within 8 min, then the column was flushed and equilibrated to starting conditions. The column oven was set to 40 °C and the autosampler was cooled to 8 °C. The untargeted HPLC-HRMS analysis was performed with an Elute HPLC system and column oven (Bruker Daltonics, Bremen, Germany) in combination with an Impact II qToF system and an Apollo II ion source (Bruker Daltonics, Bremen, Germany). Compounds were selected for fragmentation in AutoMS/MS mode (data-dependent acquisition, DDA). For targeted HPLC–MS/MS analysis, an 1260 Infinity HPLC system (Agilent, Waldbronn, Germany) was coupled to a QTRAP 6500 mass spectrometer (AB Sciex, Darmstadt, Germany) used in multiple reaction monitoring mode. Detailed mass spectrometric parameters can be found in Online Resource 1 (Tab. S1 and S2).

### Data processing and statistical analysis

Data from targeted measurements were analyzed with Sciex OS 2.2 (AB Sciex, Darmstadt, Germany) and AFB_1_-Gua and dG were quantified with external calibration. In cell culture medium, AFB_1_ was quantified with matrix-matched external calibration. MetaboScape 2024b (Version 13.0) and Data Analysis 5.3 (Bruker Daltonics, Bremen, Germany) were used for non-targeted analysis and compound identification. More parameters for feature extraction and data processing in MetaboScape can be found in Online Resource 1 (Tab. S3). Further data processing was performed in Microsoft Excel 2023 (Microsoft Corporation, Redmond, USA) and graphs were created in OriginPro 2022 (OriginLab Corporation, Northampton, MA, USA). For statistical analysis, a one-way ANOVA followed by Tukey’s post-hoc test was performed with *p* = 0.05. All experiments including only two replicates required the calculation of the range between the two samples, whereas three and more replicates required the calculation of a standard deviation.

## Results and discussion

### Key aspects of method development

The first step in method development was establishing a sample preparation method for rapid, easy and cheap extraction of DNA without losing potential analytes. The final DNA isolation procedure was based on a modified version of a previously published method for the isolation of fungal DNA (Cenis 1992). This method utilizes the distinct chemical properties of DNA in different solvents and detergents, enabling a cost-effective and straightforward isolation process. Additionally, the method does not require spin-columns with limited capacity, which are often used in DNA isolation kits. This enables scaling up for larger quantities of DNA isolation, which can be crucial for adducts formed in low yield. Nonetheless, some key points need to be taken into consideration using this method for DNA adductomics. The use of DNase-free material is mandatory to keep DNA integrity until purposely digested. After cell scraping with lysis buffer, a reduction of viscosity by aspirating and dispensing the cell lysate is crucial to highly improve protein removal. Moreover, any heating of the samples before digestion should be reduced to a minimum to avoid depurination of unstable adducts such as AFB_1_-Gua. This applies mainly to adducts binding at *N*−7 position of guanine. Furthermore, DNA precipitation after the addition of isopropanol needs to be monitored carefully to ensure complete precipitation. During precipitation, the reaction tubes can be cooled and inverted continuously to support this process. After washing with ice-cold ethanol, the solvent needs to be evaporated entirely, as remaining solvent reduces activity of later added digestion enzymes. Furthermore, alkaline phosphatase turned out to lose activity over time. To ensure its proper enzymatic activity, aside from DNA adducts, DNA bases and nucleosides also nucleotides can be included in the HPLC–MS analysis. A rise in nucleotide intensity over the other analytes is therefore attributed to the loss of alkaline phosphate activity. To check for alkaline phosphatase prior to digestion more rapidly 5-bromo-4-chloro-3-indolyl-phosphate can be used as a substrate. The blue color reaction of the solution within a few minutes confirms alkaline phosphatase activity (Jong et al. 1985).

### Metabolism in cancer cell lines

As many compounds bind to the DNA exclusively after metabolic activation, the choice of an appropriate biological system was the second challenge to be addressed. Cancer cell lines, primary hepatocytes or post-mitochondrial fractions are typical model systems for studying xenobiotic metabolism and were, therefore, compared regarding their capability to form DNA adducts. Due to cost efficiency and high availability, cancer cell lines represent a useful tool in toxicity studies, although their metabolic capacity is limited compared to primary hepatocytes (Gómez-Lechón et al. 2016). Therefore, the first experiments dealt with the induction of metabolic reactions in cancer cell lines and the comparison of cell lines from different organs to evaluate their capabilities in DNA adduct analysis.

#### Metabolic activation by β-NF preincubation

**Fig. 1:**
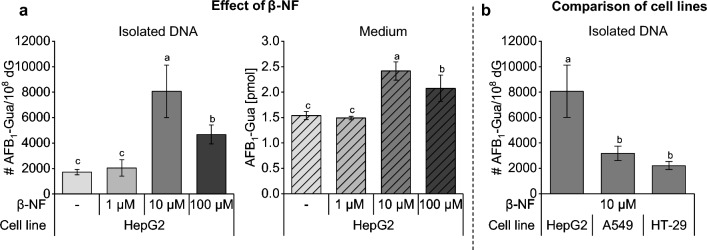
Optimization of metabolism in cancer cell lines. Effect of 1–100 µM β-naphthoflavone (β-NF) pre-treatment (24 h) on aflatoxin B_1_-guanine (AFB_1_-Gua) formation after 24 h incubation of 10 µM aflatoxin B_1_ (AFB_1_). AFB_1_-Gua was directly analyzed in the medium of HepG2 cells and in the cell lysate after DNA isolation (**a**). Comparison of DNA adduct formation in HepG2, A549 and HT-29 cells after 24 h of treatment with 10 µM AFB_1_ including 24 h pretreatment with 10 µM β-NF (**b**). Samples were analyzed via HPLC–MS/MS. The significance level was determined using a one-way ANOVA followed by Tukey’s post-hoc test with a significance threshold set at *p* = 0.05. The experiments were performed with three cell passages with two seeding replicates each (*n* = 3 × 2)

Initially, we used the cell line HepG2, because it is one of the most frequently used hepatocellular carcinoma cell lines, although it expresses CYP enzymes at relatively low levels (Westerink and Schoonen 2007a). To enhance the metabolic competence of HepG2 cells, the effect of pre-incubation with β-NF at concentrations of 1 µM, 10 µM and 100 µM was evaluated. The adduct formation of the well-known carcinogen AFB_1_ was used as a model reaction, as it correlates with CYP1A1 and CYP1A2 expression being responsible for AFB_1_ epoxidation, among other metabolic reactions (Deng et al. 2018). After DNA isolation from cell lysate and digestion, adduct formation was measured via HPLC–MS/MS. Additionally, cell culture medium was directly analyzed to cover DNA adducts excreted by DNA repair. The results are presented in Fig. 1a.

The pre-incubation of 1 µM β-NF did not affect DNA adduct formation compared to the samples without pre-incubation of β-NF, whereas 10 µM β-NF increased the amount of AFB_1_-Gua in the cell lysate approximately fourfold from 1720 ± 207 AFB_1_-Gua/10^8^ dG in the samples without β-NF pre-incubation up to 8065 ± 2057 AFB_1_-Gua/10^8^ dG after treatment with 10 µM β-NF. Similar results were obtained in the cell culture medium where 1.5 ± 0.1 pmol AFB_1_-Gua were detected in the medium without pre-treatment and 2.4 ± 0.2 pmol were detected after pre-incubation of 10 µM β-NF. A higher dose of 100 µM β-NF resulted in lower amounts of AFB_1_-Gua in both matrices compared to 10 µM β-NF, which is related to the low solubility of β-NF at this concentration in aqueous solutions.

The induction of various xenobiotic phase I metabolism enzymes by β-NF, such as CYP1A1 and CYP1A2, among others was already previously observed in HepG2 cells and the use of β-NF as an inductor of enzymes is a common method in microsomal preparations also used in DNA adduct research (Westerink and Schoonen 2007a; Hodek et al. 2013). Therefore, pre-incubation of 10 µM β-NF was used in all of our subsequent experiments to enhance metabolic activity and DNA adduct formation. Nonetheless, it needs to be considered that the increase of metabolic activity is limited to aryl hydrocarbon receptor-dependent enzymes, but further substances inducing a broader set of metabolic reactions were not included in this study.

#### Comparison of cancer cell lines

Although hepatocarcinogenic HepG2 cells metabolized AFB_1_ and enabled the detection of DNA adducts in the lysate, we also assessed the metabolic activity of other cell lines to compare the result to HepG2 cells. Xenobiotics can enter the body via lung or intestine, so that DNA adduct formation in these organs can also be an interesting topic for future studies. Therefore, DNA adduct formation in HepG2 cells was compared with lung cancer cells (A549) and colorectal carcinoma cells (HT-29). Apart from AFB_1_, which needs to be epoxidized before DNA binding, also GA, which reacts with the DNA directly, was also used for cell line comparison in preliminary experiments. As expected, DNA adduct formation after incubation of GA was comparable in all three cell lines as its binding only depends on cellular uptake and does not require metabolic activation (Fig. S3).

In contrast, DNA binding of AFB_1_ significantly differed in the three cell lines (Fig. 1b). The highest intensity of AFB_1_-Gua was reached in HepG2 cells (8065 ± 2057 AFB_1_-Gua/10^8^ dG), which was approximately threefold higher than in A549 cells (3185 ± 565 AFB_1_-Gua/10^8^ dG) and fourfold higher than in HT-29 cells (2219 ± 318 AFB_1_-Gua/10^8^ dG) (Fig. 1b). Therefore, metabolically activated HepG2 cells were used for further experiments as they exhibit the highest metabolic competence in terms of AFB_1_ epoxidation.

### Comparison of DNA adduct formation in HepG2 cells with primary hepatocytes and liver S9 mix

**Fig. 2:**
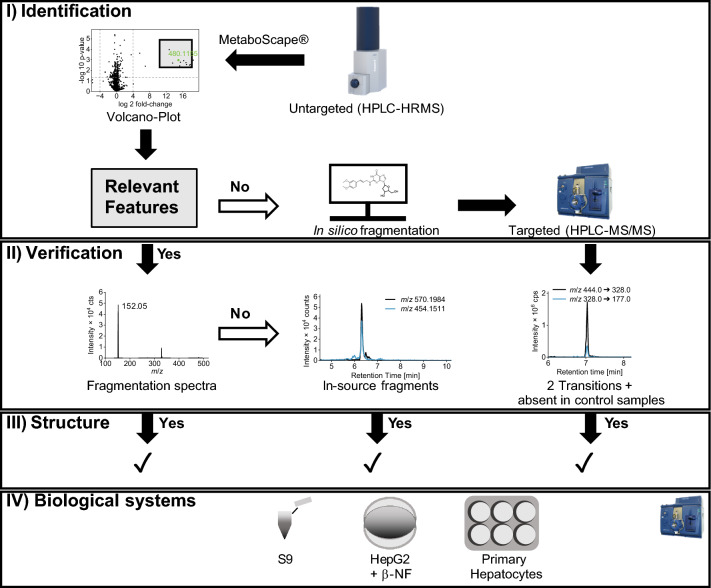
Schematic illustration of HPLC–MS-based DNA adductomics approach

The promising results, as well as low expenses in cultivation and high availability make HepG2 cells an attractive choice for fast and straightforward DNA adductomics studies. To gain a deeper understanding of their metabolic capabilities, we compared HepG2 cells with liver S9 mix and primary hepatocytes, two well-established liver model systems. Six compounds from different chemical classes, known to bind to the DNA after metabolic activation, were selected (AFB_1_, 3-MCPD, B[a]P, α-AS, β-AS and ME). Their differing metabolic requirements for the formation of the respective ultimate carcinogen enable a comprehensive comparison across the three biological systems. Along with a comparison of adduct formation, the opportunities of HPLC–MS-based adduct identification and verification are discussed in the following section. The overall approach is illustrated in Fig. 2. The results are organized by compound for easier comparison of the three biological systems.

After compound incubation, DNA extraction and digestion, DNA lysates of HepG2 cells and liver S9 fractions were initially analyzed with HPLC-HRMS, which allows for untargeted identification of DNA adducts but has limited sensitivity. Exact mass-to-charge (*m/z*) ratios and fragmentation patterns were combined with retention times (RT) into features using the software tool MetaboScape®. The most relevant features were selected based on a volcano plot, a statistical method which emphasizes the features differing the strongest between two sample groups, such as treated and control samples. The structure of the most abundant DNA adducts was elucidated by characteristic fragmentation spectra in DDA mode. The settings for precursor selection can be found in Online Resource 1 (Tab. S1). Adducts which were not selected for fragmentation can be confirmed based on predictable in-source fragmentation. If no relevant features were detected in the volcano plot, in silico fragmentation was used to create a more sensitive targeted HPLC–MS/MS method. After compound identification and verification, all samples were analyzed using the targeted HPLC–MS/MS method to compare adduct formation quantities in the three biological systems.

#### AFB_1_-Gua is mainly formed by liver S9 fraction

**Fig. 3:**
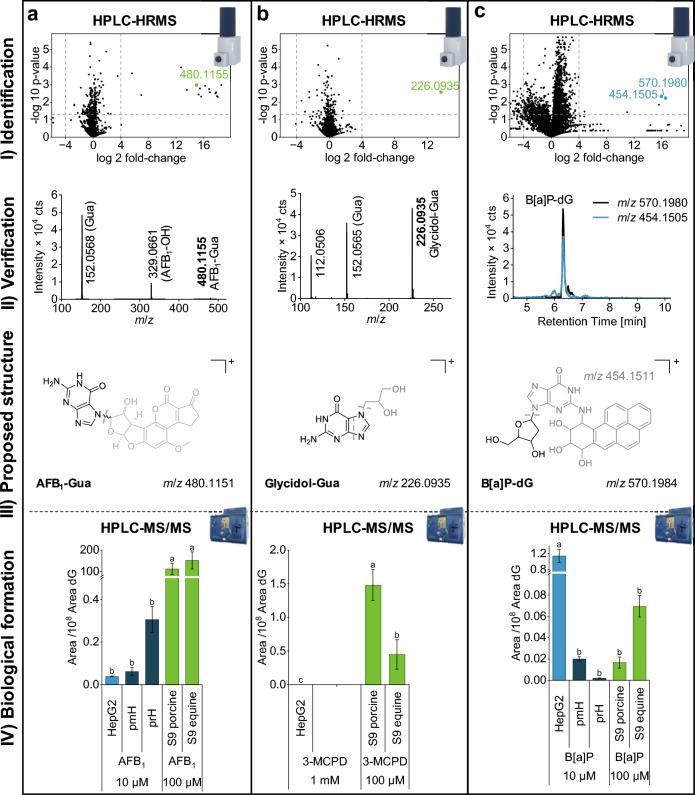
Analysis of DNA adducts of aflatoxin B_1_ (AFB_1_), 3-monochloropropanediol (3-MCPD) and benzo[a]pyrene (B[a]P). Identification of DNA adducts of AFB_1_ (**a**), 3-MCPD (**b**) and B[a]P (**c**) in digested DNA lysate using HPLC-HRMS (I). AFB_1_ and 3-MCPD data are derived from incubation of equine liver S9 mix with 100 µM, respectively. The chromatograms of B[a]P originate from incubation of HepG2 cells with 10 µM B[a]P. Chromatographic and mass spectrometric verification of identified compounds based on typical fragmentation or correlating EIC (extraction window of ± 0.01 Da) (II), proposed chemical structures (III) and comparison of adduct formation of AFB_1_, 3-MCPD and B[a]P in HepG2 cells, primary mouse and rat hepatocytes (pmH, prH) as well as porcine and equine liver S9 mix using HPLC–MS/MS. Data are normalized to underivatized dG (IV). The significance level was determined for each compound individually using a one-way ANOVA followed by Tukey’s post-hoc test with a significance threshold set at *p* = 0.05. Experiments with HepG2 cells and liver S9 mix included three replicates and primary hepatocyte experiments included two replicates

First, we compared the binding of AFB_1_ to DNA across the three biological systems. While we had access to a AFB_1_-Gua standard solution, our goal was to determine whether AFB_1_-Gua could be identified using the above-described workflow, thereby serving as a proof-of-principle. The generated volcano-plot after metabolism using equine liver S9 mix and HPLC-HRMS measurement (Fig. 3a I) yielded approximately 20 features of potential interest. These features were filtered for DNA-specific and characteristic fragments such as protonated deoxyribose (*m*/*z* 117.05), guanine (*m*/*z* 152.05) or adenine (*m*/*z* 136.06). Sorting of the features by absolute precursor intensity points towards the most relevant features. Indeed, the feature with the highest intensity (*m*/*z* 480.1155) matched the expected exact *m*/*z* 480.1151 within the usual mass accuracy of qToF instruments and was, therefore, identified as AFB_1_-Gua (Fig. 3a III). Moreover, the recorded fragmentation spectrum of this feature (Fig. 3a II) included a guanine fragment with *m*/*z* 152.0568 (calculated *m*/*z* 152.0567) and was similar to the AFB_1_-Gua fragmentation previously reported (Gerdemann et al. 2023).

Comparing the three biological systems, the AFB_1_-Gua/dG ratio was highest in both equine and porcine S9 mix enabling easy compound identification, but the adduct was also detected after treatment of HepG2 cells and primary mouse and rat hepatocytes (pmH and prH) (Fig. 3a IV). Although the induction of DNA damage by AFB_1_ has been shown previously (Yang et al. 2016), the HPLC–MS-based structural identification of AFB_1_-related DNA adducts, especially in HepG2 cells has not been reported before. The strong differences between prH and pmH are due to species differences in metabolism as discussed earlier (Gerdemann et al. 2023).

Comparing the three model systems, the signal intensity of AFB_1_-Gua is surprisingly low in primary cells compared to HepG2 cells, although AFB_1_ is efficiently metabolized by primary hepatocytes. In addition to metabolic activation, primary hepatocytes are also capable of efficient phase II detoxifying reactions, which can be related to comparably low adduct formation (Gerdemann et al. 2023). Furthermore, higher sample preparation complexity (caused by collagen sandwich culture) can lead to adduct degradation making isotopically labeled internal standards mandatory for adduct quantification. Especially *N*−7 adducts are chemically unstable (Boysen et al. 2009) resulting in subsequent depurination or cleavage of AFB_1_-dihydrodiol. Therefore, high temperature and long sample processing should be avoided to reduce adduct losses. These results are in line with high adduct contents in liver S9 fractions related to fast and simple sample preparation. Furthermore, the high AFB_1_-Gua signal in liver S9 fractions might be related to the higher incubation concentration leading to a higher amount of the reactive AFB_1_-epoxide and a more efficient DNA binding. Missing DNA repair mechanisms in S9 fractions avoid the subsequent cleavage of adducts from the DNA leading to an accumulation of derivatized DNA bases.

#### 3-MCPD forms Gua-adduct after metabolism in S9 mix

In addition to AFB_1_, we demonstrated in preliminary experiments, that the reactive 3-MCPD metabolite glycidol, effectively binds to the DNA (Fig. S2). Consequently, the next objective was to investigate whether 3-MCPD could also be activated in an in vitro system and form the same DNA adducts as glycidol.

After incubation of equine liver S9 mix with 3-MCPD, the respective volcano-plot revealed a single significant feature with a *m*/*z* of 226.0935 (Fig. 3b I) exactly matching the expected *m*/*z* of Glycidol-Gua. Also, the fragmentation to *m*/*z* 152.0565 (calculated Gua *m*/*z* 152.0567) points towards the formation of Glycidol-Gua (Fig. 3b II), which was also detected after incubation of calf-thymus DNA with glycidol (Fig S2). The short RT as well as the missing deoxyribose moiety indicate the binding position (Fig. 3b III), as deoxyribose is often cleaved during depurination in case of *N*−7 binding of compounds (Boysen et al. 2009).

Glycidol-Gua was only detected after metabolism with porcine or equine liver S9 mix but not in HepG2 cells (Fig. 3b IV). For reasons of availability, primary hepatocytes were not treated with 3-MCPD. The formation of Glycidol-Gua fits literature data, although information on the DNA adduct formation caused by 3-MCPD are still scarce and discussed critically. 3-MCPD is described to induce mutagenic and genotoxic effects in vitro especially in bacteria forming glycidol as reactive intermediate (Stolzenberg and Hine 1979; Robjohns et al. 2003). In mammalian cells, DNA damage was previously observed at high concentrations of 22.6 mM, which might be related to the formation of the non-mutagenic β-chlorolactic acid as the main mammalian metabolite (El Ramy et al. 2007; Ozcagli et al. 2016). The formation of a less toxic metabolite in mammals and a fast glutathione conjugation of the minor metabolite glycidol were also related to the lack of genotoxicity in vivo (Lynch et al. 1998; Jones 1975; Habermeyer et al. 2011). In contrast to literature data, we showed that 3-MCPD is also metabolized by mammalian liver S9 mix to a reactive, DNA binding intermediate. As the same DNA adduct was observed after incubation of calf-thymus DNA with glycidol in preliminary experiments (Fig. S2), the formation of glycidol as a reactive intermediate is most probable although it was described as a minor mammalian metabolite before. The differences were related to missing glutathione in this metabolic system, which avoided rapid detoxification and resulted in efficient DNA binding. As glutathione is known to be present in millimolar concentrations in most cell types, the observed adduct formation might be less relevant in vivo.

#### B[a]P-dG is primarily formed in HepG2 cells

B[a]P was included in the comprehensive comparison of biological systems as additional metabolic reactions are required before the formation of the ultimate carcinogen. This compound undergoes not only epoxidation but also hydrolysis, followed by a second epoxidation step before binding to the DNA (Shimada et al. 1999; Shiizaki et al. 2017).

In contrast to the two-first mentioned compounds, the adduct of B[a]P was identified in HepG2 cells treated with 10 µM B[a]P. The analysis revealed three features with a high fold-change and high significance level compared to the control samples (Fig. 3c I). Matching RT and high correlation of extracted ion chromatograms (EIC) between *m*/*z* 570.1980 and *m*/*z* 454.1505 indicated that these features belong to the same compound (Fig. 3c II). The exact *m*/*z* of 570.1980 matches the calculated *m*/*z* 570.1984 of B[a]P-dG within the typical mass accuracy of qToF instruments. This adduct has already been described previously in HepG2 cells (Shiizaki et al. 2017; Takeshita and Kanaly 2019). The difference of approximately 116.0475 Da between the two features points towards a typical cleavage of the deoxyribose unit (calculated loss of 116.0473) of the molecule and identifies the feature *m*/*z* 454.1505 as in-source fragment of B[a]P-dG (Fig. 3c III) with a calculated *m*/*z* 454.1511.

HPLC–MS/MS analysis of all samples revealed that B[a]P-dG was mainly formed in HepG2 cells, but traces were also detected in primary hepatocytes and in liver S9 mix (Fig. 3c IV). The high DNA adduct formation in HepG2 cells can be connected to a high expression of CYP1A1 and CYP1B1 caused by β-NF preincubation as well as B[a]P itself (Hockley et al. 2006; Machala et al. 2001). The formation of DNA adducts of B[a]P was very effective in HepG2 cells and has been shown previously (Takeshita and Kanaly 2019; Staal et al. 2006). The biotransformation of B[a]P in HepG2 cells was also comparable to human primary hepatocytes in a previous study demonstrating comparably high metabolic activity of HepG2 cells (Wilkening et al. 2003). The striking difference between HepG2 cells and primary hepatocytes in our study can be related to species differences and to a very efficient metabolic activation of HepG2 cells. This result underlines that not only different biological systems but also species differences in xenobiotic metabolism need to be considered in toxicological studies. Furthermore, differences in DNA repair efficiency and velocity may have a huge impact on adduct analysis in the lysate.

#### α-AS and β-AS adducts are formed in similar scale in all systems

In addition to the well-established DNA adducts mentioned above, we also investigated the formation of adducts by phenylpropanoids, which have been less extensively studied to date. As different genotoxic properties are described for α-AS and β-AS, both compounds were included in the study (Haupenthal et al. 2017; Berg et al. 2016).

**Fig. 4:**
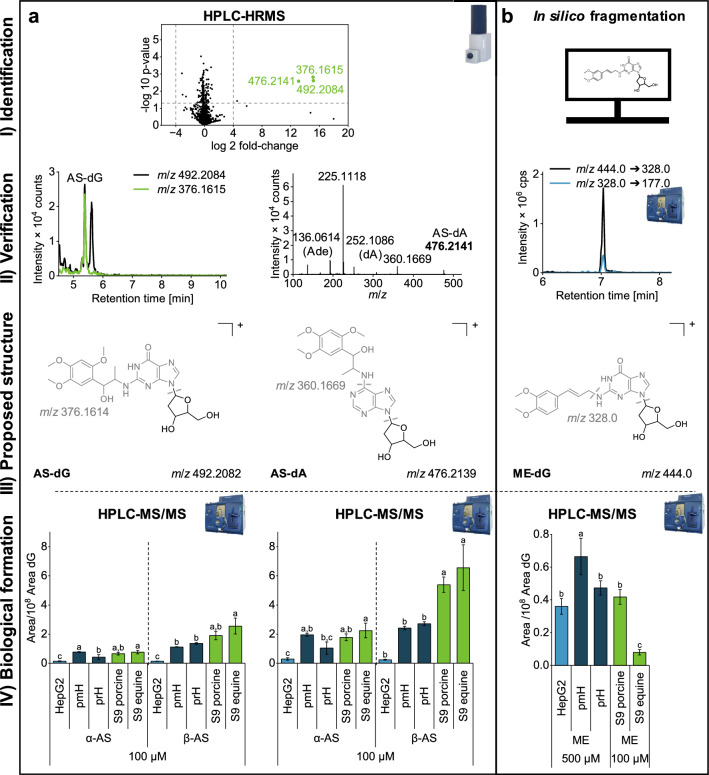
Analysis of DNA adducts of α-asarone, β-asarone (α-AS, β-AS) and methyleugenol (ME). Identification of DNA adducts α-AS, β-AS (**a**) via volcano plot and ME (**b**) after in silico fragmentation and HPLC–MS/MS analysis in digested DNA lysate (I). Chromatographic and mass spectrometric verification of identified compounds based on typical fragmentation and EIC correlation (extraction window of ± 0.01 Da) (II). Data shown in I and II were conducted from incubation of 100 µM β-AS in equine liver S9 mix or from incubation of 500 µM ME in HepG2 cells. Proposed chemical structures (III) and comparison of adduct formation in HepG2 cells, primary mouse and rat hepatocytes (pmH, prH) as well as porcine and equine liver S9 mix. The data were obtained from HPLC–MS/MS analysis after the normalization of adduct areas on underivatized dG (IV). The significance level was determined using a one-way ANOVA followed by Tukey’s post-hoc test with a significance threshold set at *p* = 0.05. Experiments with HepG2 cells and liver S9 mix included three replicates and primary hepatocyte experiments included two replicates

The incubation of 100 µM β-AS in equine liver S9 fractions yielded three features with high fold-changes and significant differences compared to the control samples in a volcano plot (Fig. 4a I). The α-AS data are shown in Online Resource 1 (Fig. S4). However, α-AS and β-AS are described to form DNA adducts upon epoxidation of the double bond eliminating stereochemical differences (Stegmüller et al. 2018). The feature with *m*/*z* 476.2141 matches the calculated m/z of AS-dA (*m*/*z* 476.2140) and the structure was verified using a fragment spectrum (Fig. 4a II right). The cleavage of a deoxyribose moiety yielded the fragment *m*/*z* 360.1669. Furthermore, *m*/*z* 252.1086 and *m*/*z* 136.0614 were assigned to the fragments dA (calculated *m*/*z* 252.1091) and Ade (calculated *m*/*z* 136.0618), respectively. Finally, the most abundant fragment with *m*/*z* 225.1118 matches the exact *m*/*z* of an oxidized asarone molecule (calculated *m*/*z* 225.1121). Altogether, this represents a characteristic fragmentation pattern for a DNA adduct and provides the most reliable option for mass spectrometric identification when reference compounds are inaccessible. A similar fragmentation was also observed in former investigations by Stegmüller et al. (2018), who published the fragmentation of 1’-OH-2H-Asaron-*N*6-dA. Main fragments were *m*/*z* 476, *m*/*z* 225, *m*/*z* 192, *m*/*z* 360, *m*/*z* 252 and *m*/*z* 136, securely confirming our results.

In contrast, adducts, which were not selected for fragmentation, can only be confirmed based on in-source fragmentation in HPLC-HRMS measurements. The features with *m*/*z* 492.2084 (RT 5.38 min) matched the theoretically calculated exact *m*/*z* of AS-dG (*m*/*z* 492.2082) within the usual mass accuracy of the qToF instrument. Because of the low intensity of this peak no fragmentation spectra were recorded, which requires a more detailed analysis of the data. Similar to the verification of B[a]P-dG two of the features had the same RT (5.38 min) and a typical difference of 116.0469 pointing towards a cleavage of a deoxyribose moiety (calculated loss of 116.0473). The high correlation of the EICs allows the first peak to be tentatively identified as AS-dG (Fig. 4a II left). These results demonstrate that theoretical EICs of typical in-source fragments such as dG, deoxyadenosine (dA) or the neutral loss of deoxyribose can be helpful in peak assignment. However, the possibility of an isomeric adduct contributing to the second peak (m/z 492.2082, RT 5.8 min) cannot be ruled out, as it was also absent in the control samples. The proposed chemical structures of the adducts are shown in Fig. 4a III.

In addition to S9 fractions, α-AS and β-AS adducts were also detected in primary hepatocytes as already described by Stegmüller et al. (2018). Furthermore, we were able to detect them in activated HepG2 cells, which was not reported previously (Fig. 4a IV). β-NF pre-incubation potentially supported adduct formation in HepG2 cells as CYP1A2, CYP3A4 and CYP2C19 are described to be responsible for epoxidation of the propenyl double bond (Cartus and Schrenk 2020). Comparing the formation quantities, β-AS formed more adducts than α-AS in primary hepatocytes and in liver S9 fractions, which correlates with the results of Stegmüller et al. (2018) in primary rat hepatocytes. In contrast, α-AS formed slightly more DNA adducts in human HepG2 cells than β-AS. The same trend was observed in comet assay using HepG2 cells as α-AS induced more strand breaks than β-AS and, therefore, appears to be more relevant for human genotoxicity (Haupenthal et al. 2017).

#### ME-dG formation is comparable in HepG2 cells and primary hepatocytes

Finally, the formation of DNA adducts by ME was analyzed, which involves phase II metabolism as well. Other than the previously mentioned compounds, the metabolism of ME involves first an oxidation of the propenyl double bond followed by a sulfation. The low stability of sulfated ME results in a loss of the sulfate group leading to a very reactive carbocation, which can bind to the DNA easily (Tremmel et al. 2017; Jeurissen et al. 2006; Herrmann et al. 2013).

In samples treated with ME, we were not able to identify any DNA adduct via HPLC-HRMS. The obtained volcano-plots from equine liver S9 mix and HepG2 cells are presented in Online Resource 1 (Fig. S5). As the adduct formation of this compound is well established (Herrmann et al. 2013; Monien et al. 2015; Schumacher et al. 2013), we tried to improve sensitivity using targeted adduct analysis. Targeted analysis is possible for DNA adduct identification without knowing the specific structure in advance, as DNA adducts consist of three specific subunits: deoxyribose + DNA base + xenobiotic. These subunits are chemically very stable, so they are suitable cleavage groups and can be used as a product ion in targeted MS analysis. Additionally, literature data on common adduct formation of compounds with comparable structures and common reactions of bioactivation prior to DNA binding help to design theoretical adducts, which can be used as a precursor. For confident identification, at least two transitions have to match in one peak or the RT needs to be verified by a reference compound. Furthermore, analyzing a control sample is mandatory to avoid false-positive results caused by matrix signals.

For the detection of ME adducts, we used transitions which have already been described in literature (Herrmann et al. 2013; Monien et al. 2015). The targeted approach revealed DNA adducts of ME (Fig. 4b) in all tested biological systems. Used transitions were the cleavage of a deoxyribose unit (*m*/*z* 444.0 → *m*/*z* 328.0) as well as the cleavage of the Gua unit from ME-Gua (*m*/*z* 328.0 → 177.0), which is formed due to in-source fragmentation (Fig. 4b III/IV). Both chromatograms show a peak with the same RT (7.1 min), which also occurred in the cell culture medium but not in the respective control samples (data not shown). The peak was therefore confirmed to belong to ME-dG. The observed fragmentation was also compared and confirmed with literature data, which reported *m*/*z* 177 and *m*/*z* 328 as fragments of ME-dG (Herrmann et al. 2013).

In contrast to most of the other compounds, ME-dG was formed in slightly higher yields in cellular systems than in liver S9 fractions (Fig. 4b IV), which might be related to the two-step bioactivation. The main enzymes involved in this metabolism are CYP1A2 and SULT1A1 (Tremmel et al. 2017; Jeurissen et al. 2006; Cartus et al. 2012). Transcription of the coding genes of these enzymes is also induced by β-NF leading to increased ME metabolism in HepG2 cells. The protein abundance of SULT1A1 in HepG2 cells is also described to be comparable to primary human hepatocytes (Westerink and Schoonen 2007b).

In previous studies, the formation of DNA adducts of phenylpropanoids was determined after incubation of the proximal carcinogen (1’-hydroxymethyleugenol), via transfected HepG2-CYP1A2 cells or in primary hepatocytes (Cartus et al. 2012; Al-Subeihi et al. 2013; Schulte-Hubbert et al. 2020). Therefore, we demonstrate the formation of the respective DNA adducts using the parent compound ME in non-transfected human cancer cells for the first time. Surprisingly, ME-dG was also formed in liver S9 fractions although the cofactor 3’-phosphoadenosine 5’-phosphosulfate (PAPS), which is necessary for sulfation, was not added. This result could, on the one hand, be related to PAPS residues in S9 preparations or, on the other hand, to a sulfation-independent adduct formation. The formation of methyleugenol-2,3-epoxide and its genotoxic properties have already been described previously (Cartus et al. 2012; Groh et al. 2012), but the formation of a DNA adduct after epoxidation of the double bound is rather unlikely, since the resulting adduct would be hydroxylated and therefore has a different *m*/*z* value.

#### Concluding comparison of biological systems

Comparing the three metabolic activation models, equine and porcine liver S9 mix produced the highest yields of DNA adducts for most of the analyzed substances. With a total amount of 2160 features extracted from the samples metabolized with equine liver S9 mix, a comparably small dataset was generated enabling easy feature identification and assignment because of few matrix signals. Furthermore, in intact cells metabolites of cytochrome P450 are formed at the endoplasmic reticulum (ER), from where they have to diffuse to the nucleus to form DNA adducts. During this passage, detoxifying phase II reactions are possible that reduce the concentrations of reactive metabolites. In contrast, S9 mix contains only a low phase II metabolizing capacity compared to intact cells, the compartmentalization of, e.g., nuclei and ER is no longer present and DNA repair processes are strongly reduced. Therefore, the here presented system with S9 and externally added DNA maximizes the formation of adducts, thereby allowing the analysis of a worst-case scenario. This property in addition to easy sample preparation, high availability and high throughput makes the use of S9 mix well-suitable especially for screening approaches and chemically unstable adducts.

HepG2 cells exhibited a lower metabolic conversion in most of the experiments, a problem which can be addressed by scaling up the culture conditions of the immortalized cell-line and improving instrument sensitivity. The cell line is also capable of DNA repair mechanisms and is, therefore, closer to the in vivo system compared to liver S9 fractions. With a total number of 7259 features, a comparably high amount of non-relevant features was extracted pointing towards more matrix signals and less purified DNA compared to liver S9 fractions. The use of volcano plots limits this issue and enables the separation of relevant and non-relevant features.

Primary hepatocytes showed comparably high metabolic activity, as all DNA adducts were formed and detected in these samples without the addition of any cofactors or metabolic activation. They represent a model system close to the in vivo situation, but low availability and high expenses in cultivation, as well as complex sample preparation, limit their suitability for DNA adductomics approaches. Furthermore, effective DNA repair mechanisms and conjugation processes reduce the time-slot to be used for DNA adduct analysis. Apart from that, the data indicate that also species differences have to be considered. However, primary human cells are very scarce and expensive. To overcome this limitation, human immortalized cell lines are commonly used. Nonetheless, differences also exist between cancer-derived cell lines and healthy cells. This highlights that in vitro models inherently involve specific compromises and careful selection of an appropriate in vitro model is crucial to obtain reliable and meaningful data.

Overall, it can be recommended to begin adduct screening using liver S9 mix, because relevant adducts are efficiently formed and the costs are relatively low. The data can be used for the subsequent development of a targeted method to detect adducts in cancer cell lines with lower metabolic activity. This approach combines low expenses in time and money and considers complete cellular systems including cell compartmentation as well as DNA repair mechanisms.

## Conclusion

The developed HPLC–MS-based method enabled detection of DNA adducts from several chemical classes (AS-dG, AS-dA, B[a]P-dG, AFB_1_-Gua, ME-dG) in a carcinogenic cell line demonstrating that immortalized cell lines are well suitable for DNA adduct analysis. The preincubation with β-NF further increased oxidative metabolism and can be used to enhance the low metabolic activity of cancer cell lines but the metabolism with liver S9 fractions reveals even higher adduct quantities for many xenobiotics. With the data from S9 fractions in hand, a very sensitive targeted HPLC–MS method can be developed to enable also detection of very low quantities formed in cancer cell lines. Also, predictable fragmentation patterns of DNA adducts can be used to verify low-abundant adduct signals. In conclusion, HepG2 cells represent a good alternative to the commonly used primary hepatocytes, because of easy availability and cultivation, simple upscaling and low costs also in combination with advanced instrumental analytical methods (Gómez-Lechón et al. 2016, 2003).

The low limit of detection as one of the most common challenges in DNA adductomics causing false negative results, was mostly overcome by the presented HPLC–MS approaches. However, it should be noted that detecting DNA adducts by the presented method does not necessarily mean that the same adducts are formed in vivo. Furthermore, care is advised when transferring these data to human and animal health, as the artificial CYP induction and the chosen cell type cannot be ruled out completely false positive results as well.

## Supplementary Information

Below is the link to the electronic supplementary material.

Supplementary file1 (DOCX 404 KB)

## Data Availability

Additional data are presented in Online Resource 1.
