# Supplementary material for: A simplified LC–MS-based method for sensitive analysis of DNA adducts utilizing accessible in vitro metabolism models
Source: Arch Toxicol. 2025 Jul 8;99(10):4021–34. doi: 10.1007/s00204-025-04125-w (PMC12454591; doi:10.1007/s00204-025-04125-w)
Supplement: Supplementary file 1 — Supplementary file1 (DOCX 404 KB) [file 204_2025_4125_MOESM1_ESM.docx]

**Supplementary material**

**for**

**A simplified LC-MS-based method for sensitive analysis of DNA adducts utilizing accessible *in vitro* metabolism models**

Andrea Gerdemann^*,1^ (andrea.gerdemann@uni-muenster.de), Matthias Behrens^1^, Georgia Günther^2^, Ahmed Ghallab^2,3^, Jan G. Hengstler^2^, Hans-Ulrich Humpf^1^, Melanie Esselen^1^

^1^Institute of Food Chemistry, University of Münster, Corrensstraße 45, 48149 Münster, Germany

^2^Leibniz Research Centre for Working Environment and Human Factors (IfADo), Ardeystraße 67, 44139 Dortmund, Germany

^3^Department of Forensic Medicine and Toxicology, Faculty of Veterinary Medicine, South Valley University, Qena 83523, Egypt

**Corresponding Author:**

*Dr. Andrea Gerdemann, Institute of Food Chemistry, 48149 Münster, Germany; e-mail address: andrea.gerdemann@uni-muenster.de

**Fig. S1:** Chemical structures of tested compounds

**Synthesis of glycidamide adducts**

Glycidamide was synthesized based on an alkaline epoxidation reaction of 0.325 mol acrylonitrile with hydrogen peroxide. The reaction product was dissolved in 50 mL DMSO leading to a calculated maximum concentration of 6.5 M in case of complete conversion. The product was not further purified as it was only used for qualitative applications. Afterwards, 100 µL of the reaction product were mixed with 100 µL of dG (1.9 mg/mL) and shaken 96 h at 37 °C in 800 µL phosphate buffer (1 M, pH 7.4). The product was also used qualitatively only and therefore not purified. As a result, concentrations are given in GA equivalents (eq) based on the starting material. The structural characterization was based on exact mass and fragmentation pattern (HPLC-HRMS) shown in Figure S1.

**Synthesis of glycidol adducts (as DNA adducts of 3-MCPD)**

DNA adducts of glycidol, which reacts with the DNA itself, were formed by incubation of 100 µL glycidol with 1.25 mg ctDNA in 900 µL phosphate buffer (1 M, pH 7.4) in a DNase-free reaction tube. After 24 h of gentle shaking at 37 °C, 500 µL of the solution were added onto a molecular filter (30 kDa, Microcon, Merck Millipore, Darmstadt, Germany) and DNA was digested enzymatically after washing. For digestion DNA was dissolved in 100 µL digestion buffer (10 mM Tris, 5 mM MgCl_2_) including 20 U DNase, 400 mU PDE and 2 U ALP. The DNA was digested overnight at 37 °C under gentle shaking. After centrifugation Glycidol-Gua was characterized based on exact mass and fragmentation pattern using HPLC-HRMS (Figure S1). As these references were also used for qualitative applications exclusively, they were not further purified.

**Fig. S2**: Fragmentation spectra of Glycidol-Gua and Glycidamide-Gua obtained after semi-synthesis and measured via HPLC-HRMS

**Comparison of cell lines**

The DNA adduct formation in different cell lines was also compared after treatment with glycidamide (GA), which reacts with the DNA directly without metabolic activation. As GA was used only qualitatively, this compound was not completely purified. The synthesis product was diluted by a factor 1000 in HBSS leading to a maximum concentration of 6.5 mM GA based on the used educt amounts for synthesis. The cells were pre-incubated 24 h with 10 µM β-naphthoflavone before addition of GA. The results are shown in Figure S4.

**Fig. S3:** Comparison of GA-Gua formation after treatment of three cell lines with glycidamide (GA). As no metabolic activation was necessary for DNA binding, the amounts of DNA adducts turned out to be very comparable

**Tab. S1:** Parameters of developed untargeted HPLC-HRMS method

**Untargeted HPLC-HRMS method**

| **Column** | Nucleodur® C18 Pyramid, 150 mm × 2 mm, 3 µm  (Macherey‑Nagel GmbH & Co. KG, Düren) | | | | | |
| --- | --- | --- | --- | --- | --- | --- |
| **Mobile Phase** | A: ACN + 0,1 % FA  B: H_2_O + 0,1 % FA | | | | | |
| **Gradient** | **Time [min]** | **Eluent A [%]** | | | **Flow Rate (mL/min)** | |
|  | 0 | 0 | | | 0.5 | |
|  | 2 | 0 | | | 0.5 | |
|  | 10 | 95 | | | 0.5 | |
|  | 12 | 95 | | | 0.5 | |
|  | 12.2 | 0 | | | 0.5 | |
|  | 15 | 0 | | | 0.5 | |
| **Column oven** | 40 °C | | |  | | |
| **Autosampler** | 8 °C | | |  | | |
| **Detection** | **HRMS (Impact II™, Bruker Daltonics, Bremen, Germany)** | | | | | |
| **Source parameter** | Ionisation | | | Elektrospray Ionization (ESI) | | |
|  | Polarity | | | Positive | | |
|  | Source Temperature | | | 300 °C | | |
|  | End Plate Offset | | | 500 V | | |
|  | Capillary Voltage | | | 4500 V | | |
|  | Nebulizer Gas | | | 3 bar | | |
|  | Dry Gas | | | 12 L/min | | |
| **MS parameter** | Mass Range | | | *m*/*z* 50-1000 | | |
|  | Spektra Rate | | | 6 Hz | | |
|  | Funnel 1 RF | | | 150 Vpp | | |
|  | Funnel 2 RF | | | 200 Vpp | | |
|  | isCID Energy | | | 0 eV | | |
|  | Hexapole | | | 50 Vpp | | |
|  | Ion Energy | | | 4 eV | | |
|  | Low Mass | | | m/z 55 | | |
|  | Collision Energy | | | 10 eV | | |
|  | Collision RF | | | 650 Vpp | | |
|  | Transfer Time | | | 60 µs | | |
|  | Pre Pulse Storage Time | | | 8 µs | | |
| **Fragmentation** | **Auto MS/MS** | | |  | | |
|  | Number of Precursor | | | 3 | | |
|  | Absolute Threshold (1000 sum) | | | 200 cts | | |
|  | Exclude after | | | 3 Spectra | | |
|  | Release after | | | 0,5 min | | |
|  | Smart Exclusion | | | 3 × | | |
|  | **Mass** | | **Isolation width** | **CE** | | **Charge** |
|  | 100 | | 4,00 Da | 15 V | | 1 |
|  | 500 | | 5,00 Da | 25 V | | 1 |
|  | 1000 | | 6,00 Da | 40 V | | 1 |

**Tab. S2:** Parameters of developed targeted HPLC-MS/MS method

**Targeted HPLC-MS/MS method**

| **Column** | Nucleodur® C18 Pyramid, 150 mm × 2 mm, 3 µm  (Macherey‑Nagel GmbH & Co. KG, Düren) | | | | | |
| --- | --- | --- | --- | --- | --- | --- |
| **Mobile Phase** | A: ACN + 0,1 % FA  B: H_2_O + 0,1 % FA | | | | | |
| **Gradient** | **Time [min]** | **Eluent A [%]** | | **Flow rate (mL/min)** | | |
|  | 0 | 0 | | 0.5 | | |
|  | 2 | 0 | | 0.5 | | |
|  | 10 | 95 | | 0.5 | | |
|  | 12 | 95 | | 0.5 | | |
|  | 12.2 | 0 | | 0.5 | | |
|  | 15 | 0 | | 0.5 | | |
| **Column oven** | 40 °C | | |  | | |
| **Autosampler** | 8 °C | | |  | | |
| **Detection** | **MS/MS (QTRAP 6500®, AB Sciex, Darmstadt, Germany)** | | | | | |
| **Source parameter** | Ionisation | | | Elektrospray ionization (ESI) | | |
|  | Polarity | | | Positive | | |
|  | Source Temperature | | | 400 °C | | |
|  | Ion Spray Voltage | | | 4500 V | | |
|  | Nebulizer Gas (GS1) | | | 45 psi | | |
|  | Heating Gas (GS2) | | | 55 psi | | |
|  | Curtain Gas | | | 35 psi | | |
| **MS parameter** | **MRM (multiple reaction monitoring)** | | | |  | |
|  | Cell Entrance Potential | | | 10 V | | |
|  | Cell Exit Potential | | | 11 V | | |
|  | Declustering Potential | | | 80 V | | |
|  | Dwell Time | | | 5 ms | | |
| **MRM  (Quantifier in bold)** | **Name** | | **Q1 [m/z]** | | **Q3 [m/z]** | **CE (V)** |
|  | **dG** | | **268.1** | | **152.1** | **15** |
|  |  | | 268.1 | | 135.0 | 50 |
|  | **dA** | | **252.1** | | **136.1** | **20** |
|  |  | | 252.1 | | 117.1 | 20 |
|  | **dC** | | 228.1 | | 112.1 | 15 |
|  |  | | **228.1** | | **95.0** | **50** |
|  | **Et-dG** | | **296.1** | | **180.1** | **15** |
|  |  | | 296.1 | | 152.1 | 40 |
|  | **8-oxo-dG** | | **284.1** | | **168.1** | **20** |
|  |  | | 284.1 | | 151.0 | 50 |
|  | **AFB_1_-Gua** | | **480.1** | | **152.1** | **25** |
|  |  | | 480.1 | | 329.1 | 25 |
|  | **GA-Gua** | | **239.1** | | **152.1** | **25** |
|  |  | | 239.1 | | 194.1 | 25 |
|  | **ME-dG** | | **444.0** | | **328.0** | **15** |
|  |  | | 328.0 | | 177.0 | 25 |
|  | **B[a]P-dG** | | **570.2** | | **454.1** | **20** |
|  |  | | 570.2 | | 152.1 | 20 |
|  | **AS-dG** | | **492.2** | | **376.2** | **20** |
|  |  | | 492.2 | | 225.1 | 20 |
|  | **AS-dA** | | **476.2** | | **225.1** | **20** |
|  |  | | 476.2 | | 360.2 | 20 |
|  | **Glycidol-Gua** | | **226.1** | | **152.1** | **20** |
|  |  | | 226.1 | | 112.1 | 20 |

**Tab. S3:** Processing parameters used for feature extraction via MetaboScape®

**MetaboScape processing parameter**

| Minimum # features for extraction  (before recursive extraction) | 2/6 |
| --- | --- |
| Presence of features in minimum # of analyses (after recursive extraction) | 3/6 |
| Intensity threshold | 10 000 cts |
| Minimal peak length | 8 |
| Feature signal | Area |
| Recursive feature extraction | Enabled |
| Retention time | 0.5-10 min |
| Mass range | m/z 10-600 |
| Missing value substitution | 1 |

**α-Asarone (equine S9)**

**Fig. S4:** Identification of DNA adducts of 100 µM α-asarone in DNA lysate of equine liver S9 mix using HPLC-HRMS

**Methyleugenol (HepG2 cells) Methyleugenol (equine S9)**

**Fig. S5:** Identification of DNA adducts of 500 µM methyleugenol in DNA lysate of HepG2 cells (left) and 100 µM methyleugenol in equine liver S9 mix (right) using HPLC-HRMS
